# Supplementary material for: Deep representation learning for clustering longitudinal survival data from electronic health records
Source: Nat Commun. 2025 Mar 14;16:2534. doi: 10.1038/s41467-025-56625-z (PMC11909183; doi:10.1038/s41467-025-56625-z)
Supplement: Supplementary file 1 — Supplementary Information [file 41467_2025_56625_MOESM1_ESM.pdf]

Deep representation learning for clustering longitudinal survival data from electronic health records

Supplementary Note 1

First, for each ICD-10 code, its subcategory (e.g. K50.1), category (e.g. K50) and block (e.g. K50-K52) were extracted using the algorithm below.

Algorithm ICD-10 mapping

```
# x is the input ICD-10 code
function ICD-10_map (x):
    if x is in block level:
        return (x, x, x)
    else if x is in category level:
        # block(x) returns the block of the ICD-10 code from a lookup table
        return (x, x, block(x))
    else:
        # category(x) returns the block of the ICD-10 code from a lookup table
        return (x, category(x), block(x))
```

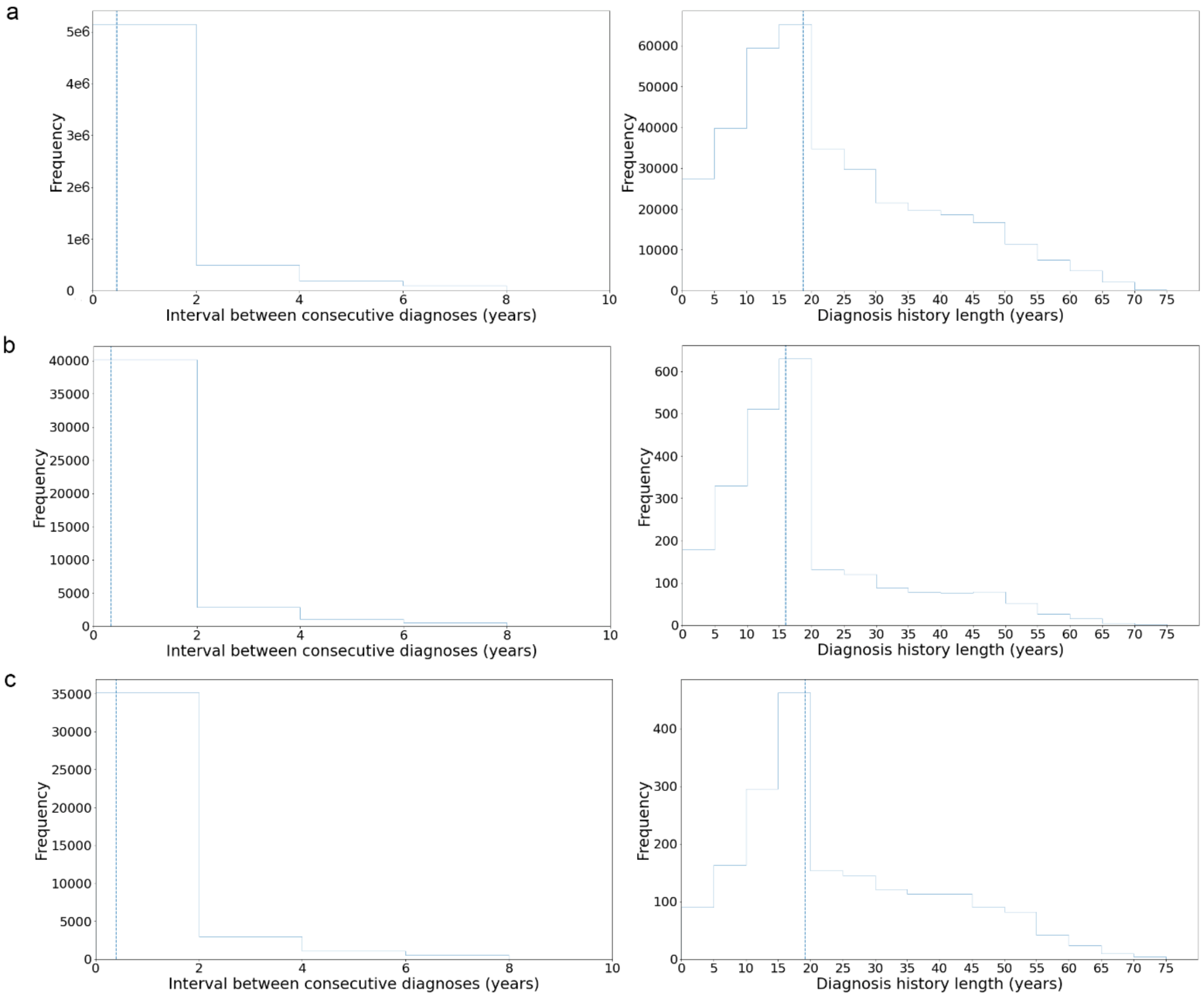

Supplementary Fig. 1: Summary of EHR data (supplementing Table 1). Interval between consecutive visits (left) and diagnosis history length (right) for **a** Overall UKBB EHR (Table 1, column 3), **b** T1D/T2D benchmark (Table 1, column 4), and **c** CD use case (Table 1, column 5).

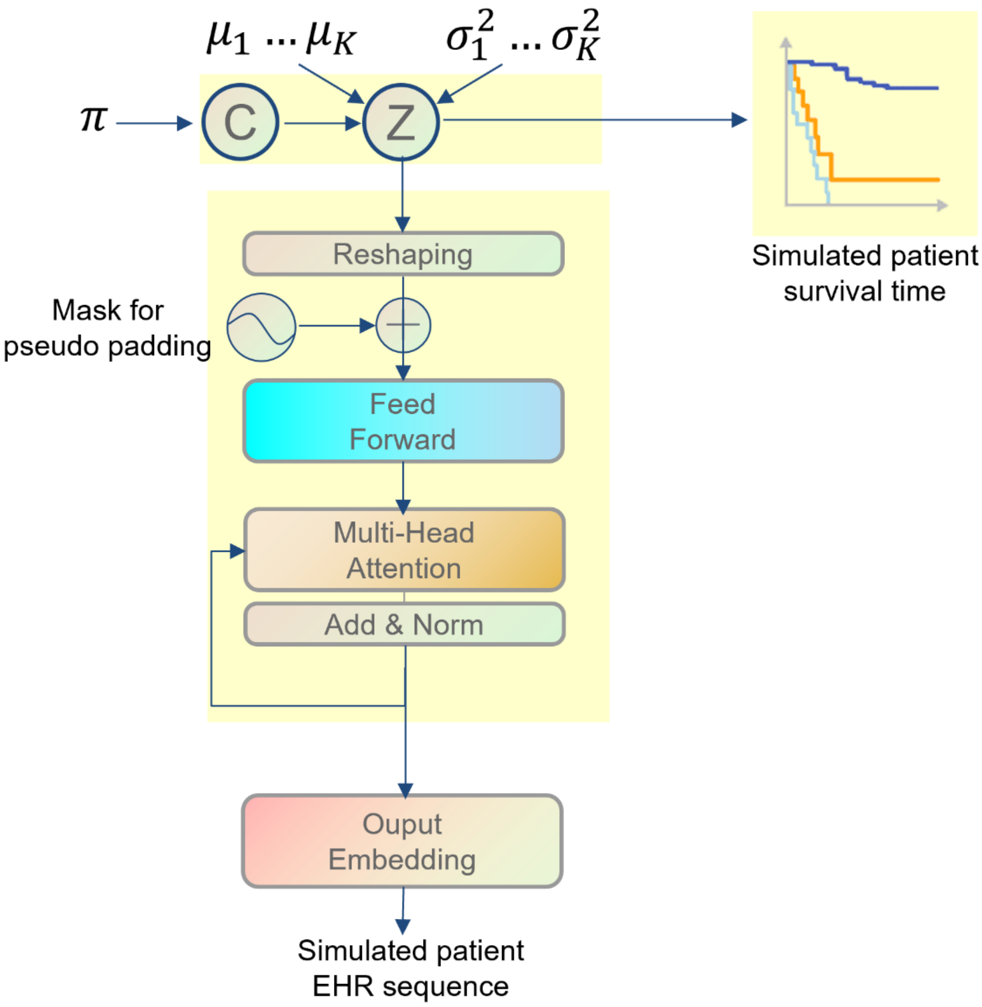

Supplementary Fig. 2: The generative process of VaDeSCEHR. For randomly generating one EHR sequence with corresponding time-to-event, we first sample a vector Z from a Gaussian mixture distribution initialized with random parameters. Z is then used to generate both a time-to-event and an EHR sequence. The time-to-event is generated by sampling from a $Weibull$ distribution. The EHR sequence is generated by feeding Z into a transformer-based decoder with randomly initialized weights and then converted to an ICD-10 code using a softmax function. For details, we refer the reader to the Methods section.

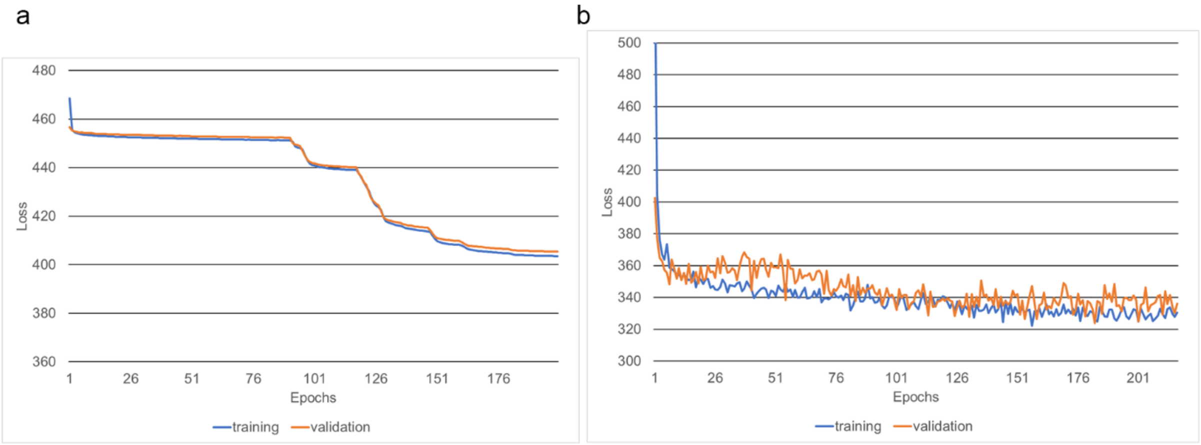

Supplementary Fig. 3: Overall loss for benchmark analysis. a synthetic benchmark. **b** T1D/T2D benchmark.

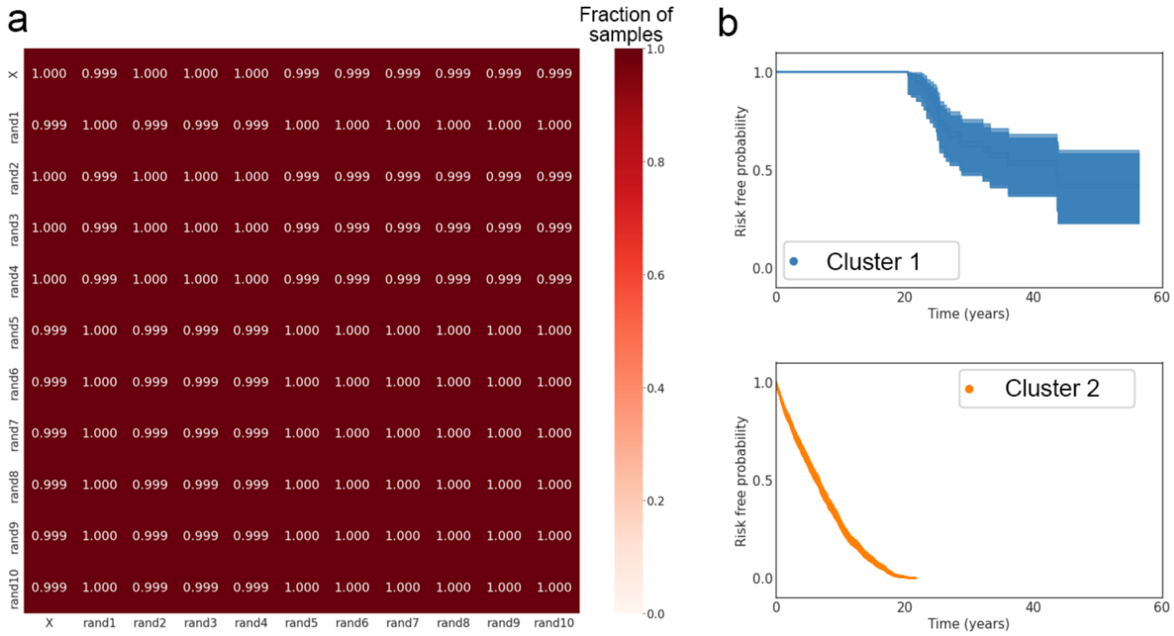

Supplementary Fig. 4: Stability analysis for T1D/T2D benchmark. Results of running the VaDeSCEHR 10 times with different initial embeddings and weight initializations (rand1-10, n =10). X indicates the result that we report as the main result in the manuscript. **a** Stability analysis for clustering. Each value indicates the fraction of the samples that obtain the same cluster assignment between two models. **b** Stability analysis for risk modeling. For each cluster, we plot the survival curve from 10 different models. Survival curves show a high degree of similarity across runs.

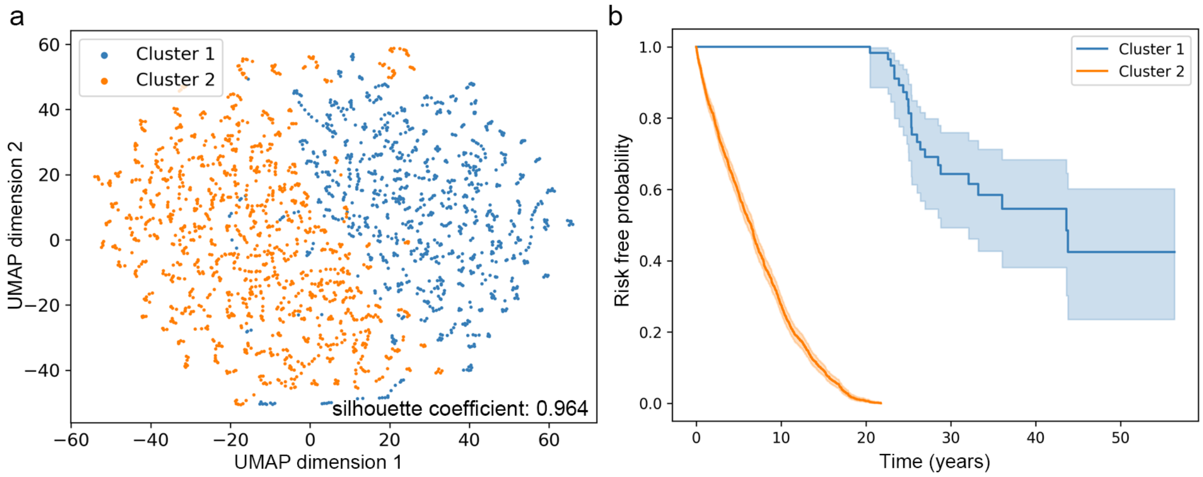

Supplementary Fig. 5: Using VaDeSCEHR to distinguish T1D from T2D patients. a UMAP of the patient representations (silhouette coefficient: 0.964). **b** Cluster-specific Kaplan–Meier curves for both clusters.

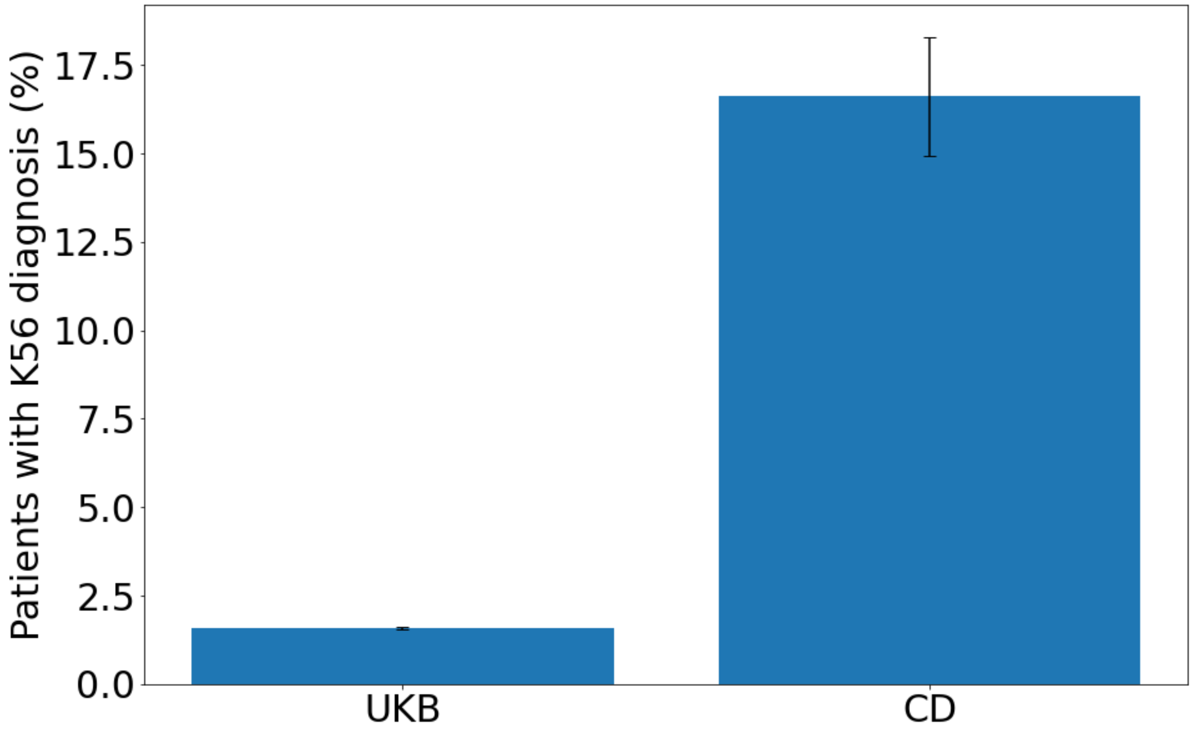

Supplementary Fig. 6: Enrichment of K56 in CD patients compared to UK Biobank patient population. Two sided Chi-squared p-value < 0.0001.

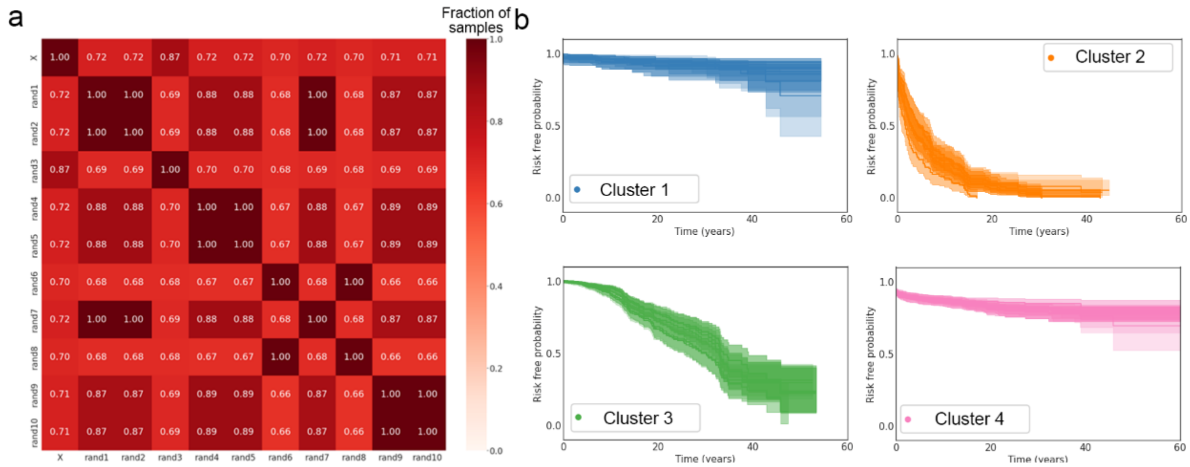

Supplementary Fig. 7: Stability analysis for the CD application. Results of running VaDeSCEHR 10 times with different initial weights and embeddings (rand1-10, n = 10). X indicates the result that we report as the main result in the manuscript. **a** Stability analysis for clustering. Each value indicates the fraction of the samples that obtain the same cluster assignment between two models. **b** Stability analysis for risk modeling. For each cluster, we plot the survival curve from 10 different models. Survival curves show a high degree of similarity across runs.

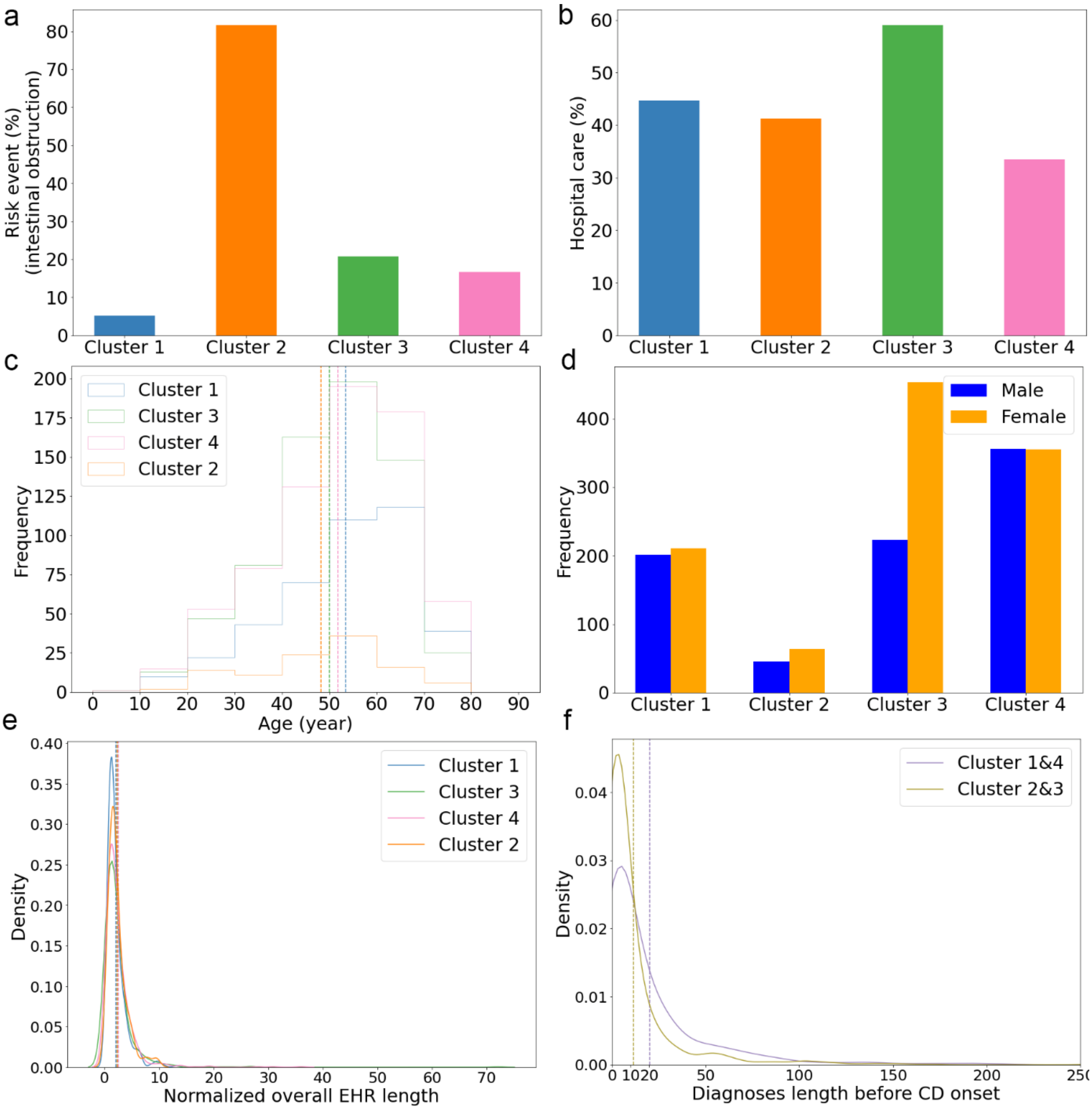

Supplementary Fig. 8: Association of CD patient subgroups with different variables. The analyses are based on 1,908 CD patients (n = 1,908). **a** Fraction of patients in each cluster with the risk event intestinal obstruction (p-value: 1.28×10^{-50}, two sided multinomial logistic regression with log likelihood ratio test, adjusting by confounding including age, sex, PC1, recruitment location, fraction of hospital care data). **b** Fraction of patients in each cluster with hospital care data (two sided Chi-squared p-value: 7.19×10^{-20}). **c** Distribution of age of onset in each cluster (two sided one-way ANOVA p-value: 6.82×10^{-05}). **d** Sex distribution in each cluster (two sided Chi-squared p-value: 2.35×10^{-10}). **e** Normalized overall diagnosis sequence length across clusters (two sided One-way ANOVA p-value: 0.19). **f** Distribution across clusters of diagnosis sequence length before first CD diagnosis (two sided one-way ANOVA p-value: 4.48×10^{-10}).

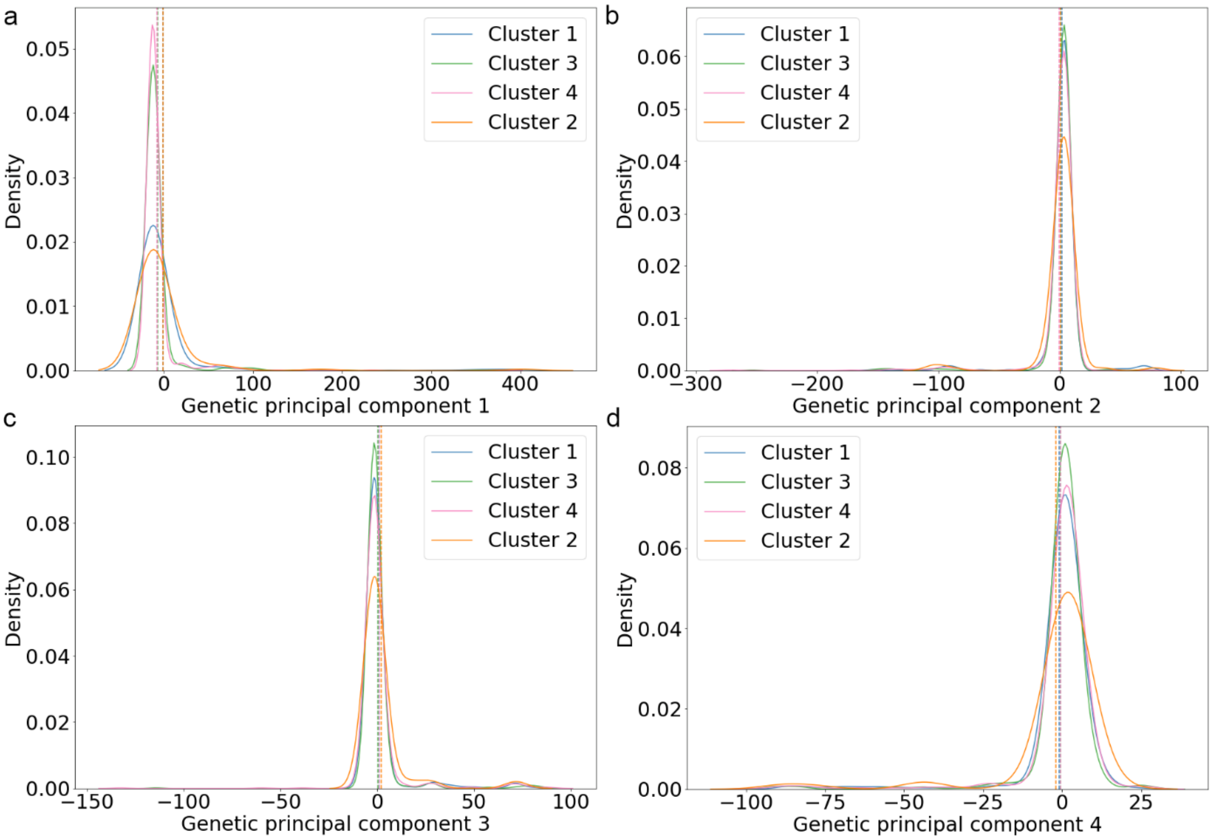

Supplementary Fig. 9: Distribution of genetic principal components (PCs) across the four CD patient subgroups. The analyses are based on 1,908 CD patients (n = 1,908). Two sided One-way ANOVA test was conducted to assess the significance of the difference between the clusters (p-value: 0.01, p-value: 0.29, p-value: 0.56, p-value: 0.64 for PC 1, 2, 3, and 4 respectively).

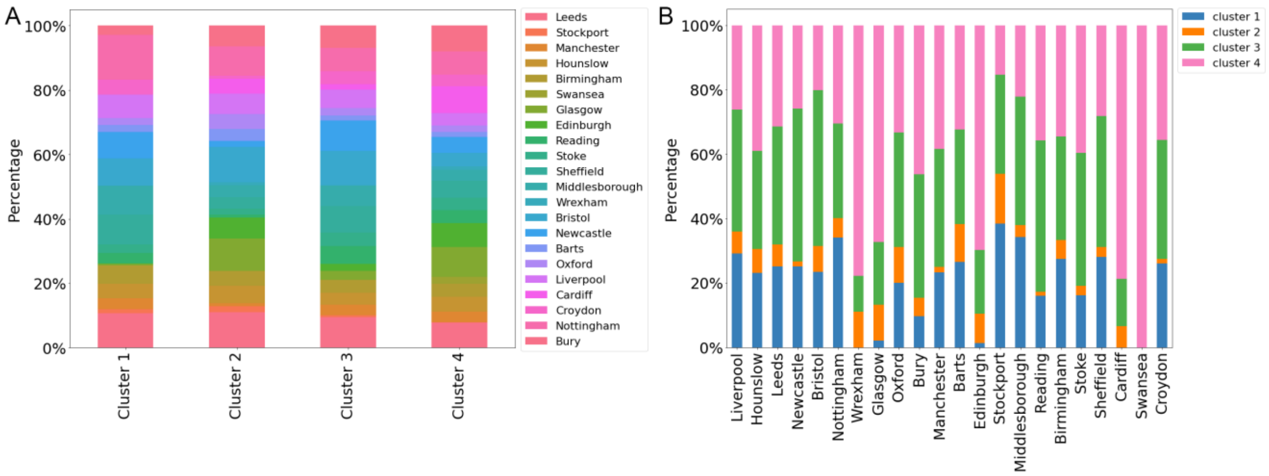

Supplementary Fig. 10: Association of CD patient subgroups with location of UKB recruitment. The analyses are based on 1,908 CD patients (n = 1,908) (permutation chi-squared p-val = 1). **a** proportion of locations in each cluster. **b** proportion of clusters in each location.

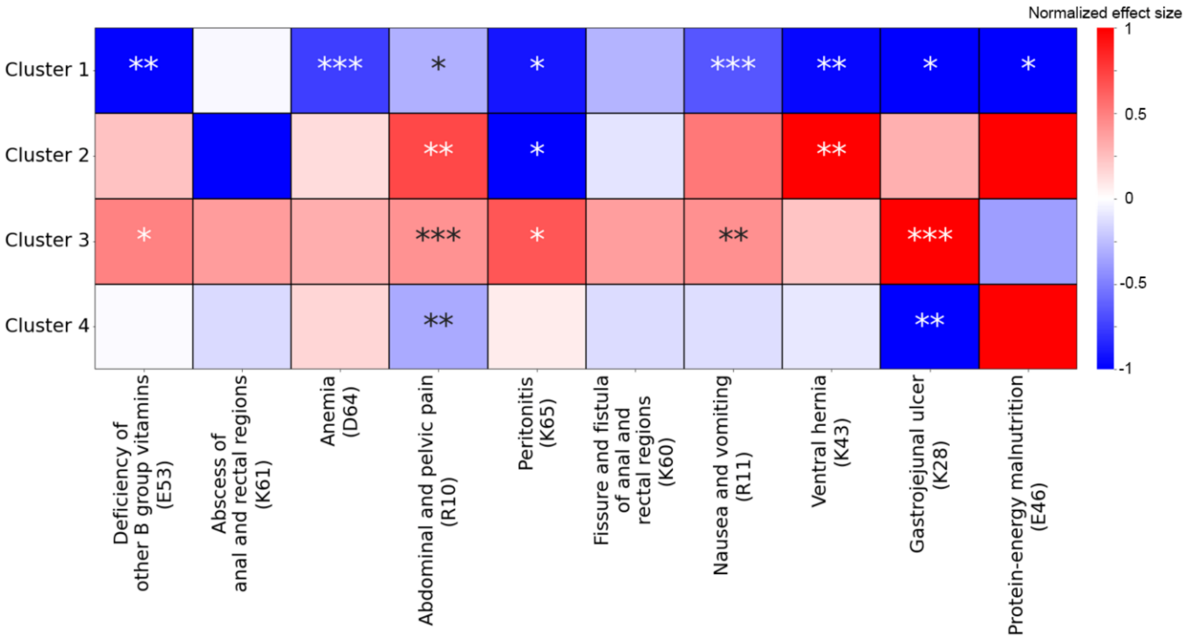

Supplementary Fig. 11: Association of CD clusters with individual diagnoses. The analyses are based on 1,908 CD patients (n = 1,908). Cluster-specific enrichment of individual CD-relevant diagnoses, computed relative to all other clusters, ranging from blue (negative) to red (positive) association. Asterisks indicate the significant level, adjusting p-values with the aim of controlling the false discovery rate (FDR) using the Benjamini-Hochberg procedure: * adjusted p-value < 0.05, ** adjusted p-value < 0.01 and *** adjusted p-value < 0.001.

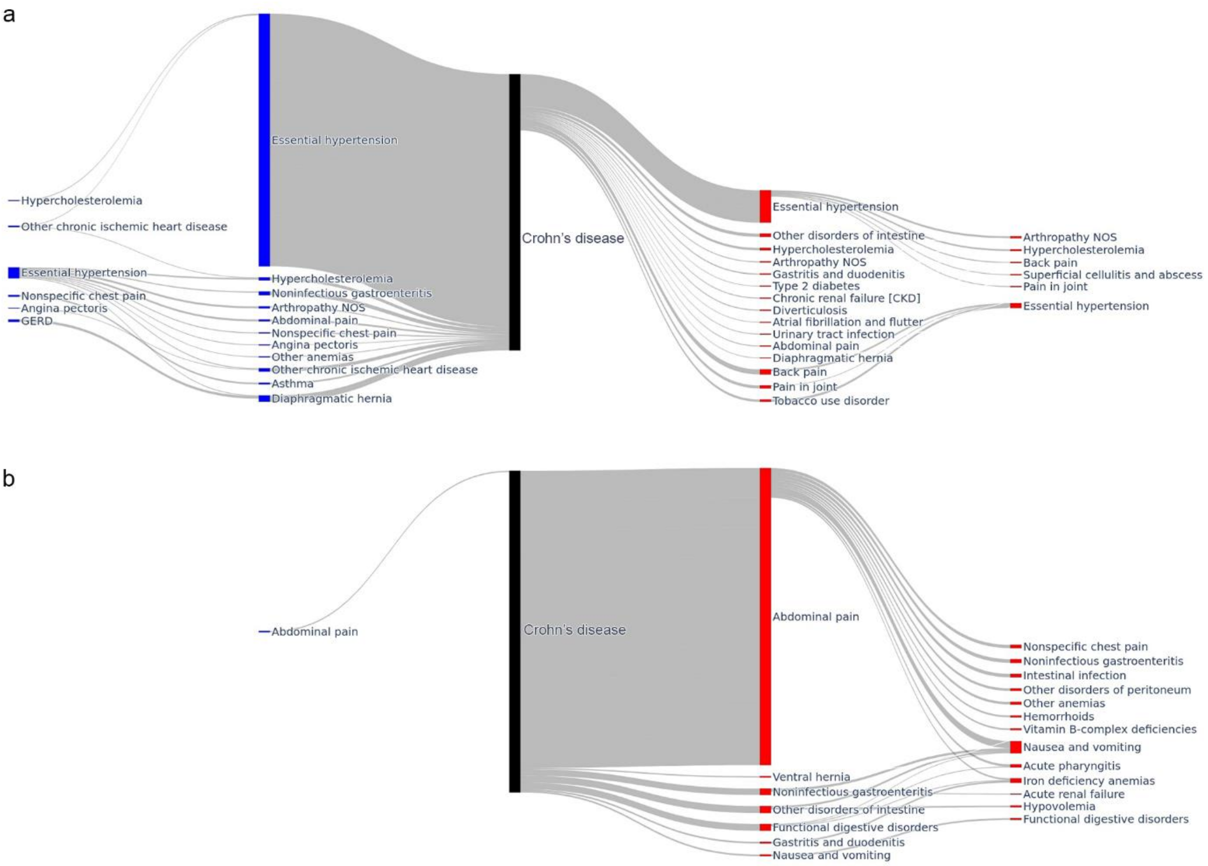

Supplementary Fig. 12: Enrichment of diagnosis subsequences across fast and slowly progressing clusters. Diagnoses are colored by occurrence before (blue) or after (red) the first CD diagnosis (black). **a** Diagnosis subsequences significantly enriched in slow progressors (clusters 1 and 4) relative to fast progressors (clusters 2 and 3) (logistic regression coefficient > 0 and adjusted p-value < 0.05) with the width of the band representing the number of patients with a given subsequence. **b** Diagnosis subsequences significantly enriched in fast progressors but not in slow progressors (logistic regression coefficient < 0 and adjusted p-value < 0.05).

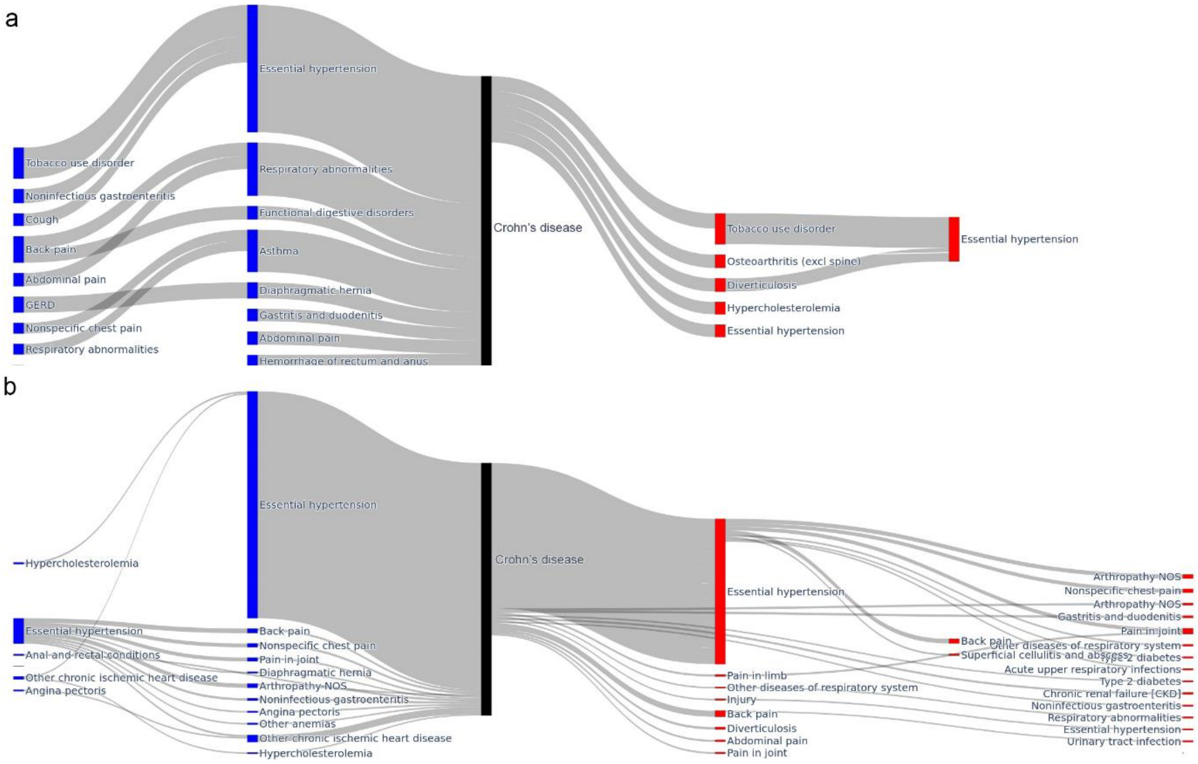

Supplementary Fig. 13: Association of CD clusters with diagnosis subsequences. Diagnosis subsequences enriched in cluster 1 **a** relative to cluster 4 **b** (logistic regression with a significance threshold at p-value: 0.05).

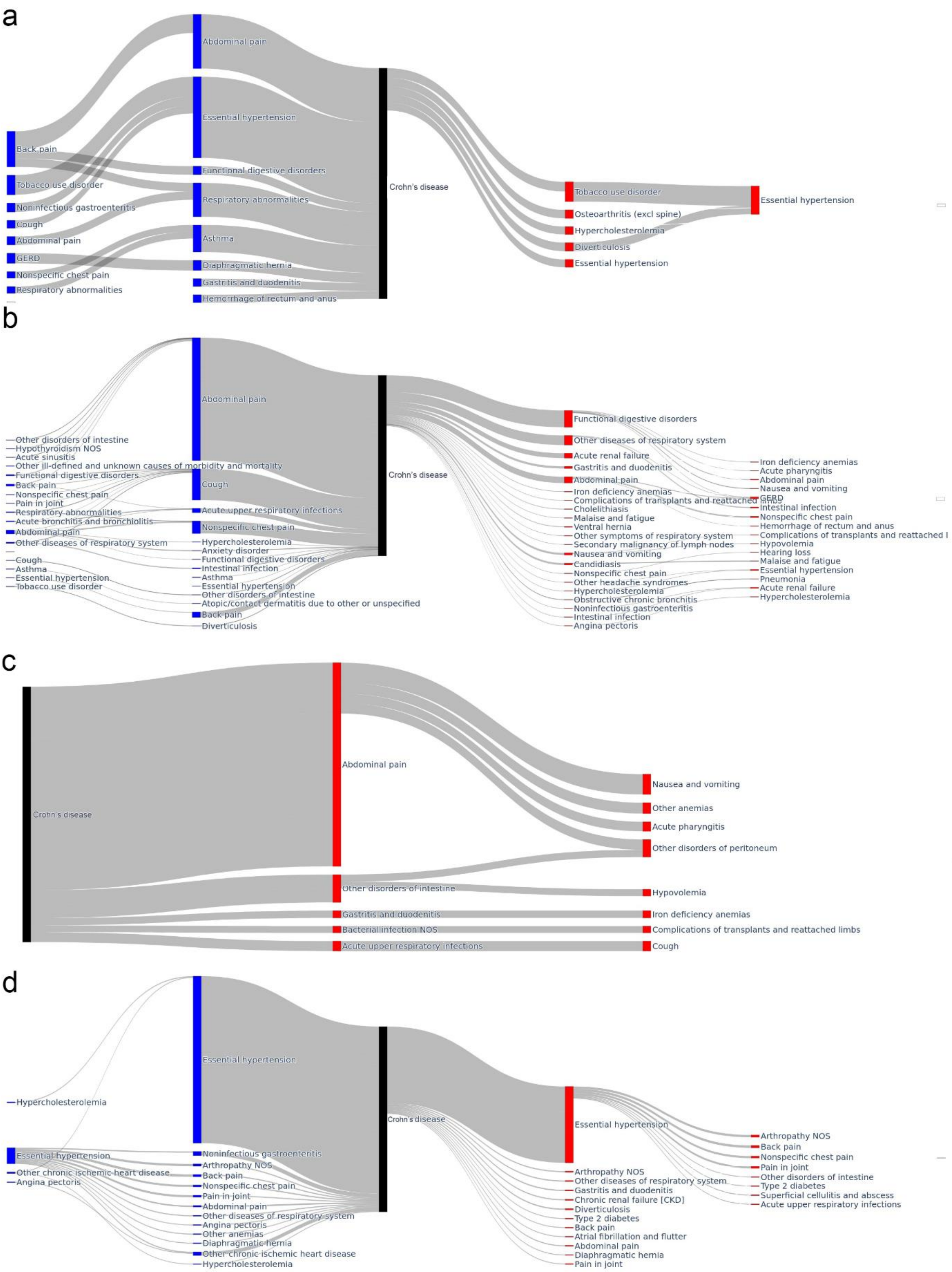

Supplementary Fig. 14: Differentially enriched diagnosis subsequences per cluster. a cluster 1, **b** cluster 2, **c** cluster 3, and **d** cluster 4. Statistical significance of association is determined using logistic regression (p-value < 0.05).

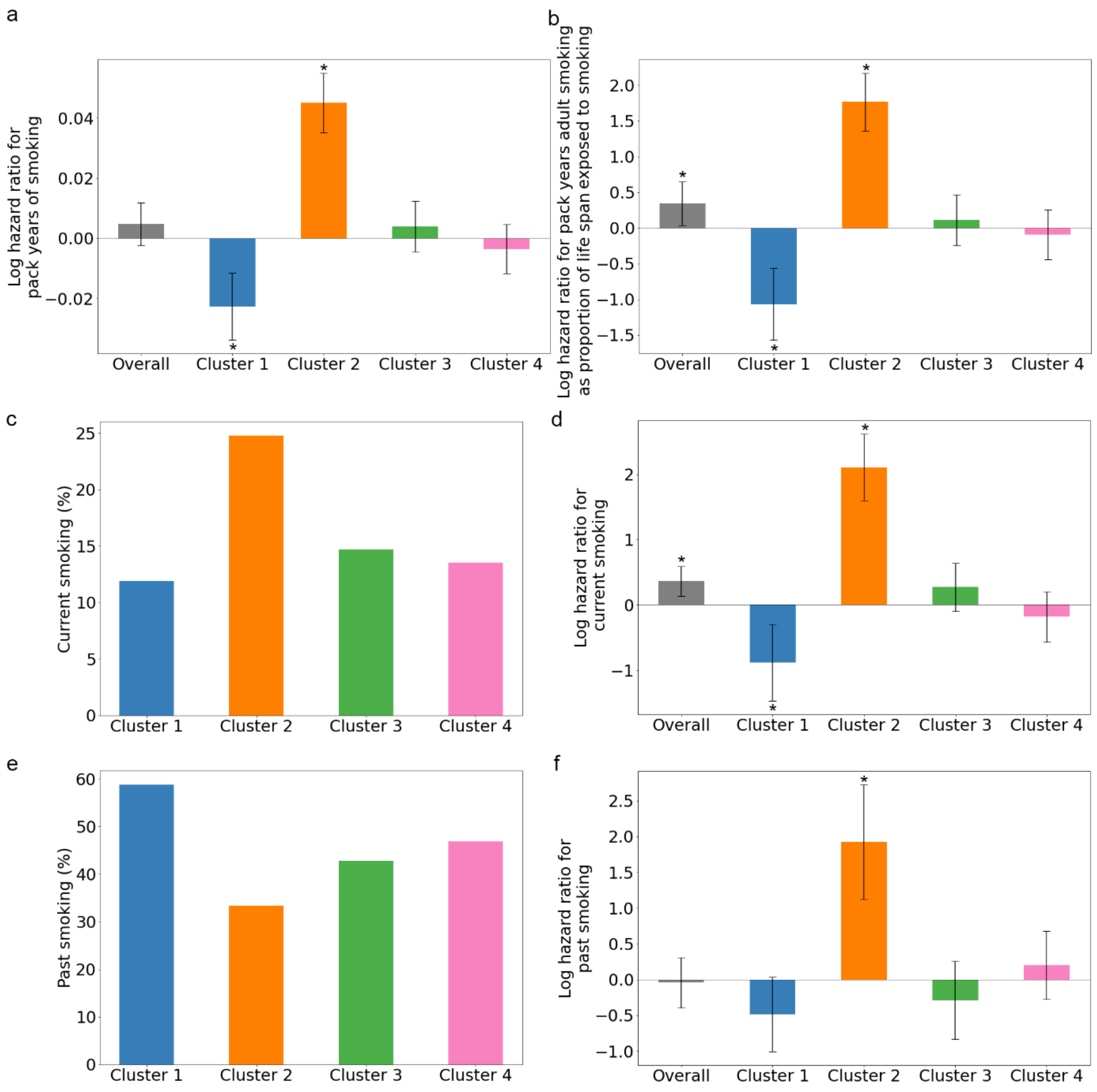

Supplementary Fig. 15: Association of CD clusters with smoking behavior. The analyses are based on 1,908 CD patients (n = 1,908). **a** Association of pack years of smoking (UK Biobank data field: 20161) with risk of intestinal obstruction (p-values are Overall: 0.19, Cluster 1: 7.17×10^{-05}, Cluster 2: 4.16×10^{-19}, Cluster 3: 0.37, Cluster 4: 0.39). **b** Association of pack years adult smoking as proportion of life span exposed to smoking with risk of intestinal obstruction (UK Biobank data field: 20162) (p-values are Overall: 0.03, Cluster 1: 3.40×10^{-05}, Cluster 2: 1.02×10^{-17}, Cluster 3: 0.55, Cluster 4: 0.59). **c** Percentage of current smokers (p-value: 0.038, multinomial logistic regression with log likelihood ratio test). **d** Association of current smoking with risk of intestinal obstruction (p-values are Overall: 0.002, Cluster 1: 0.003, Cluster 2: 6.56×10^{-16}, Cluster 3: 0.15, Cluster 4: 0.35). **e** Percentage previous smokers (p-value: 2.39×10^{-9}, multinomial logistic regression with log likelihood ratio test). **f** Association of past smoking with risk of intestinal obstruction (p-values are Overall: 0.81, Cluster 1: 0.07, Cluster 2: 2.56×10^{-6}, Cluster 3: 0.30, Cluster 4: 0.41). The error bars represent the 95% confidence intervals and * represents the significance of the association as determined using two sided multivariate Cox regression (p-value < 0.05).

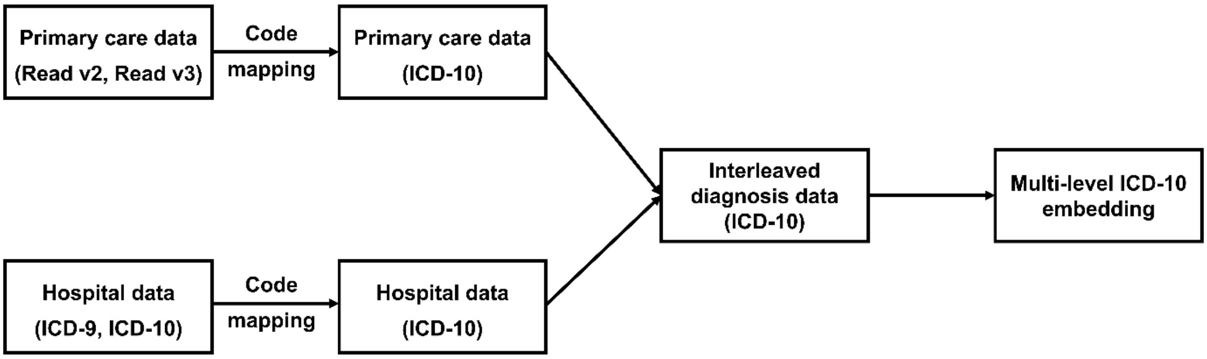

Supplementary Fig. 16: UKB EHR data parser pipeline. First, we map the various types of available diagnosis codes to ICD-10. Then we interleave the primary and hospital diagnoses based on their time stamps. Finally, we generate the multi-level ICD-10 embedding (details are described in the Method section).

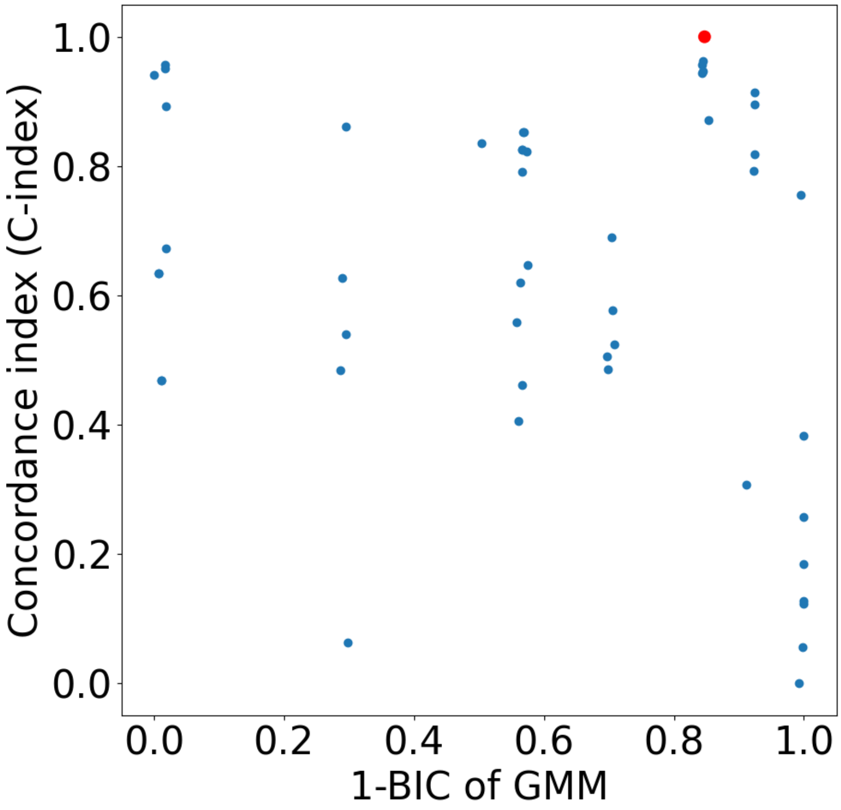

Supplementary Fig. 17: Hyperparameter optimization for the CD application. The number of clusters $\{1, 2, \ldots, K\}$ was optimized jointly with the other hyperparameters. The best combination of hyperparameters (including the number of clusters) was determined by encouraging a low Bayesian information criterion (BIC) and a high concordance index (CI) through maximizing: $\sqrt{CI^2 + (1 - BIC_{norm})^2}$, where the BIC was normalized to the interval [0, 1]. The red dot represents the hyperparameter combination finally being selected with a cluster number of 4, which corresponds to the results of CD use case we describe in the main text. Details of the hyperparameters are shown in Supplementary Table 4.

Supplementary Table 1: Summary of the data sets used in this study (supplementing Table 1)

		UK Biobank EHR	T1D/T2D benchmark	CD application
**Location**	Glasgow	18644	98	41
	Middlesborough	21283	108	106
	Cardiff	17875	75	67
	Leeds	44185	175	158
	Bristol	43000	149	213
	Newcastle	36995	135	221
	Nottingham	33870	167	122
	Bury	28311	123	134
	Reading	29399	81	90
	Birmingham	25493	84	121
	Liverpool	32799	103	197
	Manchester	13937	60	108
	Sheffield	30381	135	116
	Barts	12574	34	63
	Oxford	14054	45	58
	Croydon	27363	73	182
	Hounslow	28866	82	172
	Edinburgh	17193	76	33
	Stoke	19426	68	88
	Stockport	3791	13	23
	Swansea	2280	15	6
	Glasgow	18644	98	41

Supplementary Table 2: Trials run as part of Bayesian hyperparameter optimization for the pretraining of transformer encoder.

trials	hidden_size	num_attention_h	num_hidden_laye	intermediate_size	initializer_range	dropout	learning_rate	weight_decay	loss
1	384	12	9	2048	0.05	0.2	0.001	1.00E-06	1.33867
2	384	8	12	1024	0.04	0.2	1.00E-05	0.0001	0.99953
3	768	12	9	1024	0.02	0.3	1.00E-06	1.00E-05	1.21922
4	768	16	9	1024	0.02	0.1	0.0001	0.0001	0.54278
5	768	16	12	1280	0.01	0.5	0.0001	0.01	0.89176
6	384	12	9	1280	0.04	0.1	0.001	1.00E-06	1.10544
7	384	16	12	2048	0.02	0.1	1.00E-05	0.0001	0.87461
8	384	8	3	1024	0.04	0.1	0.001	0.1	0.85247
9	768	12	6	1280	0.02	0.2	0.0001	0.1	0.64979
10	384	16	9	1024	0.02	0.2	1.00E-05	1.00E-05	0.99285
11	768	8	6	1024	0.02	0.2	0.0001	0.0001	0.68340
12	384	16	12	1024	0.05	0.3	0.0001	1.00E-05	0.83419
13	768	16	6	1280	0.03	0.2	1.00E-05	0.0001	1.00722
14	384	12	9	1024	0.01	0.1	1.00E-06	0.001	1.04359
15	768	16	3	2048	0.01	0.1	1.00E-06	0.001	1.21871
16	768	12	3	1280	0.01	0.3	0.0001	0.001	0.48211
**17**	**768**	**16**	**6**	**1280**	**0.02**	**0.2**	**0.0001**	**0.001**	**0.41522**
18	768	12	6	1280	0.03	0.1	0.0001	0.1	0.41944
19	768	16	3	2048	0.03	0.3	0.0001	0.01	0.48143
20	768	8	3	2048	0.03	0.3	0.0001	0.01	0.46234

Supplementary Table 3: Trials run as part of Bayesian hyperparameter optimization for the fine-tuning of CD use case.

trials	num_cluster	dropout_ prob	epoch	latent_dim	learning_ rate	num_reshape _layers	weibull _shape	weight_decay	Value
1	2	0.4	50	5	1.00E-05	2	1	0.001	1.007
2	2	0.4	50	5	0.001	2	1	0.001	1.016
3	2	0.3	50	20	5.00E-05	4	4	0.001	0.692
4	2	0.1	100	5	1.00E-05	2	1	0.001	1.007
5	2	0.1	100	5	0.001	2	1	0.001	1.070
6	2	0.3	100	5	0.001	4	1	0.001	1.032
7	2	0.5	100	20	0.001	1	3	0.0001	0.789
8	2	0.2	150	5	1.00E-05	2	1	0.0001	1.000
9	2	0.2	150	10	0.0005	3	3	1.00E-05	1.220
10	2	0.5	150	15	5.00E-05	1	3	0.0001	0.881
11	2	0.2	150	15	0.0005	4	4	1.00E-05	0.911
12	2	0.1	150	20	0.001	3	3	0.0001	0.973
13	2	0.3	200	15	0.0001	4	3	1.00E-05	0.861
14	2	0.2	200	20	1.00E-05	2	2	0.01	0.731
15	2	0.3	200	20	0.0005	4	3	1.00E-05	0.837
16	2	0.4	300	5	1.00E-05	2	1	1.00E-06	0.992
17	2	0.4	300	5	0.001	3	5	1.00E-06	1.249
18	2	0.4	300	15	0.0001	4	3	1.00E-05	0.850
19	3	0.4	50	5	0.0001	4	5	1.00E-05	1.287
20	3	0.1	50	20	0.001	1	3	0.0001	0.690
21	3	0.1	50	20	0.001	2	3	0.0001	0.700
22	3	0.4	100	5	0.0001	4	5	1.00E-05	1.288
23	3	0.3	150	5	1.00E-05	3	4	0.0001	1.217
24	3	0.3	150	5	1.00E-05	4	4	0.0001	1.219
25	3	0.1	150	15	1.00E-05	1	4	0.01	0.976

26	3	0.2	200	5	0.0005	3	2	0.001	1.234
27	3	0.1	200	5	0.0005	4	3	0.001	1.300
28	3	0.4	300	10	0.0001	2	3	0.001	0.986
29	3	0.3	300	20	0.0001	4	2	1.00E-06	0.563
30	3	0.4	300	20	0.0001	4	2	1.00E-06	0.573
31	4	0.3	50	10	0.0001	3	4	0.0001	1.002
32	4	0.5	100	10	1.00E-05	1	2	0.0001	0.865
33	4	0.4	100	10	0.0001	2	4	1.00E-05	1.025
34	4	0.5	100	15	1.00E-05	1	1	1.00E-06	0.616
35	4	0.1	100	20	5.00E-05	2	1	0.01	0.468
36	4	0.2	100	20	1.00E-05	1	2	1.00E-06	0.673
37	4	0.5	100	20	0.0005	1	2	0.01	0.634
38	4	0.3	150	5	5.00E-05	4	5	1.00E-05	1.281
39	4	0.4	150	10	0.0001	2	4	0.0001	0.962
40	4	0.3	200	5	5.00E-05	4	5	1.00E-05	1.275
41	4	0.5	200	5	5.00E-05	4	5	0.0001	1.268
42	4	0.4	200	10	5.00E-05	3	3	1.00E-05	1.001
43	4	0.1	200	20	1.00E-05	1	3	0.01	0.956
44	4	0.3	250	5	5.00E-05	4	5	1.00E-05	1.266
45	4	0.5	250	15	1.00E-05	1	5	1.00E-06	0.911
46	4	0.1	250	20	1.00E-05	4	4	0.01	0.893
**47**	**4**	**0.4**	**300**	**5**	**0.0005**	**2**	**5**	**1.00E-06**	**1.311**
48	4	0.1	300	15	0.001	2	1	1.00E-05	0.305
49	4	0.4	300	20	1.00E-05	1	2	0.001	0.952
50	4	0.1	300	20	5.00E-05	1	5	1.00E-06	0.941
