# Supplementary material for: Deep representation learning for clustering longitudinal survival data from electronic health records
Source: Nat Commun. 2025 Mar 14;16:2534. doi: 10.1038/s41467-025-56625-z (PMC11909183; doi:10.1038/s41467-025-56625-z)
Supplement: Supplementary file 2 — Description of Additional Supplementary Files [file 41467_2025_56625_MOESM2_ESM.pdf]

Description of Additional Supplementary Files

Supplementary Data 1: Results of pathway PRS. Significance assessed using logistic regression with log likelihood ratio test, which was a two-sided test. And multiple testing corrected using the Benjamini–Hochberg procedure.
